# Antimicrobial Activity of a Repurposed Harmine-Derived Compound on Carbapenem-Resistant *Acinetobacter baumannii* Clinical Isolates

**DOI:** 10.3389/fcimb.2021.789672

**Published:** 2022-01-24

**Authors:** Anke Breine, Mégane Van Gysel, Mathias Elsocht, Clémence Whiteway, Chantal Philippe, Théo Quinet, Adam Valcek, Johan Wouters, Steven Ballet, Charles Van der Henst

**Affiliations:** ^1^ Microbial Resistance and Drug Discovery, Vlaams Instituut voor Biotechnologie-Vrije Universiteit Brussel (VIB-VUB) Center for Structural Biology, Vlaams Instituut voor Biotechnologie (VIB), Flanders Institute for Biotechnology, Brussels, Belgium; ^2^ Structural Biology Brussels, Vrije Universiteit Brussel (VUB), Brussels, Belgium; ^3^ Namur Medicine and Drug Innovation Center (NAMEDIC), University of Namur (UNamur), Namur, Belgium; ^4^ Research Group of Organic Chemistry, Departments of Chemistry and Bioengineering Sciences, Vrije Universiteit Brussel, Brussels, Belgium; ^5^ Research Unit in the Biology of Microorganisms (URBM), NARILIS, University of Namur (UNamur), Namur, Belgium; ^6^ Laboratory of Evolutionary Genetics and Ecology, URBE, University of Namur (UNamur), Namur, Belgium; ^7^ Molecular Biology and Evolution, Universite´ Libre de Bruxelles (ULB), Brussels, Belgium

**Keywords:** *Acinetobacter baumannii*, carbapenem-resistant, Gram-negative, pathogenic bacteria, repurposed compound, drug screening

## Abstract

**Objectives:**

The spread of antibiotic resistant bacteria is an important threat for human health. *Acinetobacter baumannii* bacteria impose such a major issue, as multidrug- to pandrug-resistant strains have been isolated, rendering some infections untreatable. In this context, carbapenem-resistant *A. baumannii* bacteria were ranked as top priority by both WHO and CDC. In addition, *A. baumannii* bacteria survive in harsh environments, being capable of resisting to disinfectants and to persist prolonged periods of desiccation. Due to the high degree of variability found in *A. baumannii* isolates, the search for new antibacterials is very challenging because of the requirement of drug target conservation amongst the different strains. Here, we screened a chemical library to identify compounds active against several reference strains and carbapenem-resistant *A. baumannii* bacteria.

**Methods:**

A repurposing drug screen was undertaken to identify *A. baumannii* growth inhibitors. One hit was further characterized by determining the IC_50_ and testing the activity on 43 modern clinical *A. baumannii* isolates, amongst which 40 are carbapenem-resistant.

**Results:**

The repurposing screen led to the identification of a harmine-derived compound, called HDC1, which proves to have bactericidal activity on the multidrug-resistant AB5075-VUB reference strain with an IC_50_ of 48.23 µM. In addition, HDC1 impairs growth of 43 clinical *A. baumannii* isolates.

**Conclusions:**

We identified a compound with inhibitory activity on all tested strains, including carbapenem-resistant clinical *A. baumannii* isolates.

## Introduction

The rise of antibiotic resistant bacteria is a global threat for healthcare, making it possible to succumb to diseases that were previously treatable (Wong et al., 2017). This has been acknowledged by both the World Health Organization (WHO) and the Centers for Disease Control and Prevention (CDC), which generated a list of drug-resistant pathogens for which new antibiotics are urgently needed (World Health Organization, 2017; CDC, 2019). The top priorities of these lists are antibiotic-resistant *Acinetobacter baumannii* bacteria (Whiteway et al., 2021).

*A. baumannii* is a Gram-negative, opportunistic bacterium, belonging to the ESKAPE group (*Enterococcus faecium, Staphylococcus aureus, Klebsiella pneumoniae, Acinetobacter baumannii, Pseudomonas aeruginosa* and *Enterobacter* spp.) of the most problematic nosocomial pathogens (Rice, 2008; Santajit and Indrawattana, 2016; Whiteway et al., 2021). While *A. baumannii* is a ubiquist (i.e. it can be found in soil, on human skin and in water sources), its presence especially imposes a threat in clinical settings (Vallenet et al., 2008; Wieland et al., 2018). This is due to a remarkable combination of resistance capabilities of *A. baumannii*, which is able to persist prolonged periods of desiccation, resist to disinfectants and to acquire drug resistance at a high rate (Da Silva and Domingues, 2016; Zeidler and Müller, 2019). Infections caused by *A. baumannii* commonly occur in immunocompromised patients and manifest as ventilator-assisted pneumonia, bacteremia and to a lesser extent skin or urinary tract infections (Weiner et al., 2016; Whiteway et al., 2021). Treatment of these infections becomes increasingly difficult, as multidrug-resistant, extensively drug-resistant or even pandrug-resistant strains have been reported, with the latter being resistant to all available antibiotics, including carbapenems (Magiorakos et al., 2012; Gallagher et al., 2015). An important hurdle in the development of new antimicrobials against *A. baumannii* is the high diversity found between isolates, leading to a still open pan-genome (Adams et al., 2008).

In this context, the discovery of new inhibitory molecules, active against multidrug-resistant *A. baumannii* strains, is therefore crucial (World Health Organization, 2017; CDC, 2019). Harmine and harmine derivatives are β-carbolines that present pharmacological effects including anti-inflammatory, neuroprotective, antidiabetic, and antitumor activities (Zhang et al., 2020). β-carbolines also show anti-infective potential (Faheem et al., 2021). For natural and synthetic β-carboline analogs, antibacterial, antifungal, antiviral, antimalarial, antileishmanial and antitrypanosomal properties have been reported. Most studies deal with biological evaluation of the molecules and, only in some rare cases, potential mechanisms of action have been proposed. Mechanistic studies demonstrated that harmine effectively repressed the HSV-2 induced upregulation of Interleukin-1β (IL-1β), Tumor Necrosis Factor-α (TNF-α), Interleukin-6 (IL-6), and Interleukin-8 (IL-8) (Chen et al., 2015). Mechanistically, substituted β-carbolines exhibit their anti-infective properties *via* several targets, including Topoisomerase II, DNA gyrase, MAPK, PfHsp90 or trypanothione reductase (Faheem et al., 2021).

For example, it has been shown that harmine exerts antileishmanial activity both *in vitro* and *in vivo* and causes necrosis by a nonspecific membrane damage in *Leishmania donovani* promastigotes (Lala et al., 2004). Harmine inhibits *P. falciparum* heat shock protein 90 by specific competition with its ATP-binding domain (Shahinas et al., 2012). Harmine analogs were further studied as binders to *P. falciparum* heat shock protein 90 (PfHsp90) and two compounds inhibited the parasite *in vitro* at micromolar concentrations, reducing parasitemia, and prolonging the survival of *P. berghei*-infected mice (Bayih et al., 2016). Adequate substitution of the β-carboline scaffold led to enhanced nematicidal effect (Xia et al., 2019). Manzamine alkaloids, which are complex natural compounds consisting of a β-carboline nucleus, have been isolated and found to exhibit potent anti-infective activities by inhibiting target kinases like GSK-3β and MtSK (Ashoka et al., 2021).

9-substituted harmine derivatives show an inhibitory effect on dengue virus and impair the maturation and release of virus particles to the extracellular medium affecting the spreading of the infection (Quintana et al., 2016). Their mode of action still needs to be clarified. 9N-methylharmine neither affects viral adsorption-internalization events nor viral RNA synthesis. The antiviral activity is not related to the ability of the compound to downregulate p38 MAPK phosphorylation.

Harmine derivatives with 7,9- or 2,7,9-substituted 7-oxy-1-methyl-β-carboline scaffolds inhibit activity of SerB2, an essential metabolic enzyme and suspected virulence factor of *Mycobacterium tuberculosis* (Frédérick et al., 2012; Carvalho et al., 2017; Carvalho et al., 2020; Pierson et al., 2020).

In this paper, we aimed at the discovery of a compound active against most clinical isolates. We performed a repurposing screen on a compound library, which led to the identification of a harmine-derived compound, called HDC1, with inhibitory activity on the growth of all the tested clinical isolates, amongst which 40 are carbapenem-resistant.

## Material and Methods

### Compound Library and Synthesis of HDC1

A compound library of the Namur Medicine & Drug Innovation Center (NAMEDIC) was provided for a growth inhibition screen against *A. baumannii*. All compounds were dissolved in 100% DMSO and used for the initial screen at 100 µM. The active compound HDC1 (1-methyl-2-benzyl-7-benzyloxy-9-benzyl-β-carbolin-2-ium bromide) was synthesized as previously described (Frédérick et al., 2012) with the following optimizations: 1-methyl-7-hydroxy-β-carboline was synthesized by adding 1-methyl-7-methoxy-β-carboline (0.600 g, 2.83 mmol, 1 equiv.), hydrobromic acid (12 ml, 48% in H_2_O) and acetic acid (12 ml) into a round-bottom flask, equipped with reflux condenser. The mixture was refluxed overnight under argon atmosphere and subsequently added to distilled water (100 ml). The precipitate was isolated *via* filtration, washed with cold water and dried under vacuum yielding 1-methyl-7-hydroxy-β-carboline with 81% (0.454 g) yield. ^1^H-NMR (500 MHz, DMSO-d6) δ (ppm): 12.59 (s, 1H), 10.62 (s, 1H), 8.39-8.29 (m, 2H), 8.21 (d, J = 8.7 Hz, 1H), 7.03 (d, J = 1.7 Hz, 1H), 6.89 (dd, J = 8.7, 1.7 Hz, 1H), 2.94 (s, 3H). Next, 1-methyl-2-benzyl-7-benzyloxy-9-benzyl-β-carbolin-2-ium bromide was synthesized. First 1-methyl-7-hydroxy-β-carboline (0.500 g, 2.52 mmol, 1 equiv.) was dissolved in anhydrous N,N,-dimethylformamide (20 ml) into a flame-dried microwave vial under argon atmosphere. Then KOtBu (0.849 g, 7.57 mmol, 3 equiv.) was added and the mixture was stirred for 30 minutes at room temperature. Subsequently benzyl bromide (3.00 ml, 25.2 mmol, 10 equiv.) was added and the mixture was heated overnight at 75°C. Afterwards the crude mixture was filtered, and the precipitate was washed with CH_2_Cl_2_. The volatiles in the filtrate were removed under reduced pressure and the crude product was subjected to column chromatography (cyclohexane/ethyl acetate) yielding the desired product with 46% (0.638 g) yield. ^1^H-NMR (500 MHz, DMSO-d6) δ (ppm): 8.86 (d, J = 6.6 Hz, 1H), 8.72 (d, J = 6.6 Hz, 1H), 8.49 (d, J = 8.8 Hz, 1H), 7.57 (d, J = 2.1 Hz, 1H), 7.50-7.46 (m, 2H), 7.40-7.25 (m, 9H), 7.23 (dd, J = 8.8, 2.1 Hz, 1H), 7.12 (d, J = 7.2 Hz, 2H), 6.99 (d, J = 7.2 Hz, 2H), 6.02 (s, 2H), 5.99 (s, 2H), 5.27 (s, 2H), 2.85 (s, 3H). ^13^C-NMR (126 MHz, DMSO-d6) δ (ppm): 162.8 (Cq), 148.0 (Cq), 139.6 (Cq), 137.5 (Cq), 136.2 (Cq), 135.4 (Cq), 134.7 (Cq), 133.5 (Cq), 129.1 (CH), 129.0 (CH), 128.5 (CH), 128.3 (CH), 127.5 (CH), 126.6 (CH), 125.4 (CH), 124.9 (CH), 114.7 (CH), 113.7 (CH), 112.8 (Cq), 95.0 (CH), 70.1 (CH2), 59.8 (CH2), 48.3 (CH2), 16.0 (CH3). The spectroscopic data were in accordance with those reported by Frédérick et al. (2012).

### Strains, Media and OD_600nm_ Measurements

Taking into account the high genomic dynamics observed for *A. baumannii* bacteria, we used the proposed modern nomenclature in the field  (Gallagher et al., 2015) by renaming the sub-cultured isolates by adding “-VUB” to the strain name, although these strains are *a priori* identical or very similar to the ones provided by the National Reference Center (NRC) Laboratory for Antibiotic-Resistant Gram-Negative Bacilli (CHU UCL Namur, Yvoir, Belgium). All *A. baumannii* reference strains and clinical isolates were cultured at 37°C in LB broth media under agitation (355 cpm) conditions for 16h before subsequent experiments. For IC_50_ determination, bacterial cells corresponding to OD_600nm_=0.1 were transferred to a 96 well flat bottom plate (Greiner, Austria) containing varying concentrations of HDC1 (0.1; 1; 10; 25; 50; 75; 100 and 1000 µM) in LB broth media with 1% DMSO. For determination of the activity of HDC1 on the 43 clinical *A. baumannii* isolates, bacterial cells corresponding to OD_600nm_=0.1 were transferred to a 96 well flat bottom plate (Greiner, Austria) containing 100 µM of HDC1 in LB broth media with 1% DMSO. For both experiments, the positive control included bacterial cells corresponding to OD_600nm_=0.1 of the used strains in LB broth media with 1% DMSO. The negative control included both LB broth media and LB broth media supplemented with 1% DMSO. Collection of data was done using the Cytation 1 (BioTek, United States). The OD_600nm_ absorbance of all bacterial cultures was measured every 30 min for 24h at a temperature of 37°C and agitation of 355 cpm (cycles per minute). In all analysis, growth inhibition is defined as decreasing absorbance values over time compared to the positive control. The classification of strains into inhibition levels is based on the ratio of the final absorbance value of the HDC1-treated bacteria (OD_600nm; HDC1_) compared to the final absorbance value of the non-treated control (OD_600nm; control_) as followed: (i) low inhibition (OD_600nm; HDC1_ > 50% of OD_600nm; control_); (ii) intermediate inhibition (10% of OD_600nm; control_ < OD_600nm; HDC1_ < or = 50% of OD_600nm; control_) and (iii) complete inhibition (OD_600nm; HDC1_ < or = 10% OD_600nm; control_). The data were measured in biological triplicate.

### CFU Determination

After absorbance measurements for IC_50_ determination, the positive control (AB5075-VUB grown in LB with 1% DMSO) and AB5075-VUB grown in presence of 100 µM HDC1, were resuspended in PBS, brought to the same OD_600nm_ and plated on LB agar plates in appropriate dilutions. After 16 h incubation of the LB agar plates at 37°C, CFUs were counted to assess any bacteriostatic or bactericidal effect. All data was measured in biological triplicate. To estimate the initial bacterial load, the relationship between the absorbance at OD_600nm_ and CFUs was determined for the strain AB5075-VUB. This was done by plating serial dilutions of bacterial cultures with a known OD_600nm_ on LB agar and counting the corresponding CFUs (see **Supplementary Table 1**).

**Figure:**
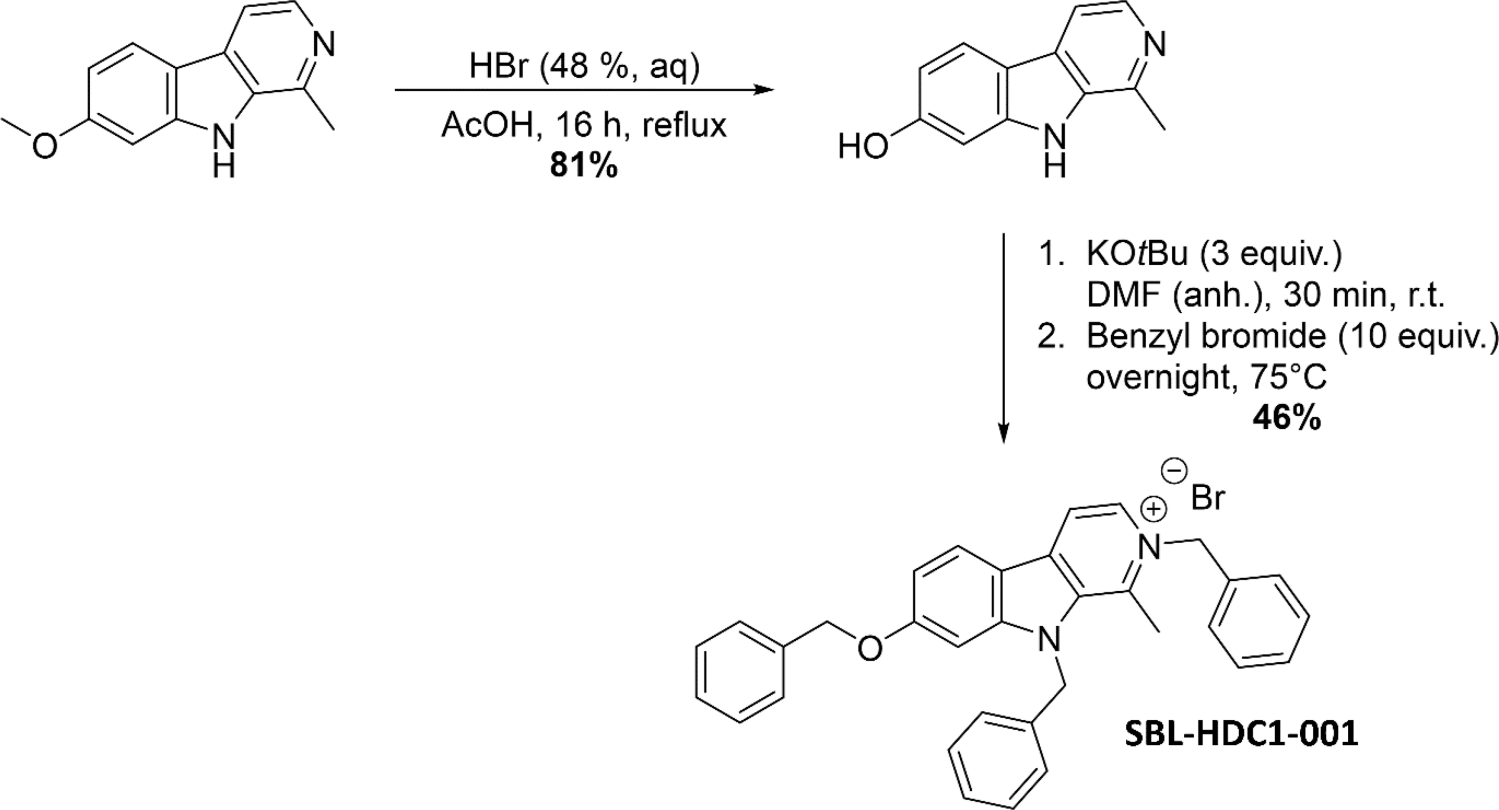


### IC_50_ Calculation

For the determination of the IC_50_, GraphPad Prism 9 (GraphPad Software, LLC) was used. After 20 hours, the OD_600nm_ absorbance kinetic data were obtained and normalized using the positive control absorbance value as 100% viability and the absorbance value of 1 mM HDC1 as 0% viability. The analysis was then done on the normalized data by nonlinear regression curve fitting. The IC_50_ value is shown with a 95% confidence interval (CI).

### Statistical Analysis

All data shown are represented as mean ± standard deviation of three biological replicates, except otherwise stated. CFU were statistically analyzed by an unpaired t-test. All growth curves were statistically analyzed by a Mann Whitney test. The p values < 0.05 were considered significant.

## Results

### Repurposing Screen Reveals Compound With Inhibitory Activity on AB5075-VUB

The initial screen from a chemical library of the Namur Medicine & Drug Innovation Center from the University of Namur (UNamur) aimed at the identification of growth inhibitors for problematic multidrug-resistant *A. baumannii* strains. The strain initially used for this screen is a multidrug-resistant *A. baumannii* reference strain, AB5075-VUB. This strain is a derivative from the parental strain AB5075 that was clonally isolated in our laboratory at the VUB (Vrije Universiteit Brussel).

**Figure 1:**
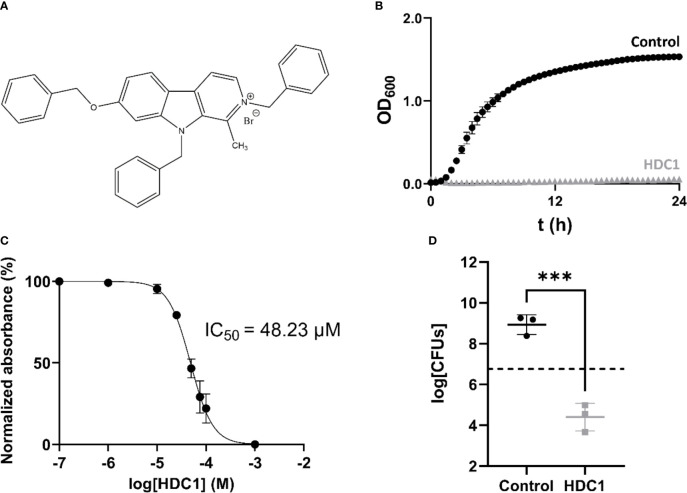
**(A)** Structure of HDC1. **(B)** Growth curve of AB5075-VUB in presence (grey triangles) and absence (black spheres) of 100 µM of HDC1. **(C)** Non-linear regression curve of normalized absorbance reads in function of compound concentration. **(D)** Number of viable bacteria after 24 h incubation without and with 100 µM of HDC1. The initial bacterial load is represented by the dashed line on the plot. All data points in this figure are shown as mean ± standard deviation of three independent biological replicates. ***p < 0.001.

The screen showed complete growth inhibition by one compound called HDC1, for it is a harmine-derived compound (**Figure 1**). HDC1 was originally synthesized for an anticancer drug screen. Interestingly, compounds with anticancer activity have lately been explored as potential antimicrobials (Cheng et al., 2019). In line with this tendency, HDC1 was further characterized for other activities.

### HDC1 Has Bactericidal Activity on AB5075-VUB

To determine the potency of the compound, the minimum concentration required for 50% growth inhibition, IC_50_, was determined for the AB5075-VUB strain. The analysis showed that HDC1 has an IC_50_ of 48.23 µM (95% CI 44.76-51.83) (**Figure 1C**). To determine the potential antimicrobial effect of HDC1 on AB5075-VUB viability, the strain was grown in presence of 100 µM of HDC1. After 24 h incubation, the bacteria were resuspended in fresh media without the compound and plated on LB agar plates for CFUs enumeration. After 24h incubation, the number of recovered bacteria is significantly different when bacteria are incubated with the compound compared to the control group (**Figure 1D**). While a 40-fold increase in CFUs is observed for the control group, a 1000-fold decrease of CFUs is observed in the presence of HDC1, compared to the initial bacterial inoculum. This shows a significant bactericidal activity of HDC1 on the multidrug-resistant AB5075-VUB reference strain.

### HDC1 Has Broad Inhibitory Activity on All the Tested Clinical Isolates

*A. baumannii* has a highly dynamic genome (Wright et al., 2016). The presence of mobile genetic elements and the efficient acquisition of genes through horizontal gene transfer are not only responsible for the pathogen’s success in obtaining drug resistance and environmental persistence, but they are also the reason isolates have become more and more diverse (Adams et al., 2008; Imperi et al., 2011; Wright et al., 2016). The core genome of the pathogen’s strains is reported to be relatively small and the accessory genome of strains can be up to 25-46% unique (Adams et al., 2008; Imperi et al., 2011). This heterogeneity found in isolates renders the search for antimicrobial compounds increasingly difficult. It is therefore important for a new potential antimicrobial to exert its activity not only on a few *A. baumannii* strains, but on a multitude of diverse and clinically relevant isolates.

To determine the activity of HDC1 on recent isolates, we used 43 recent *A. baumannii* clinical isolates, amongst which 40 are carbapenem-resistant strains (Valcek et al., 2021). In addition to these recent clinical strains, clonal isolates of three frequently used reference strains were also included. Two of these strains, ATCC19606 and ATCC17978, are older type strains, compared to the more recent and multidrug-resistant AB5075 reference strain (Gallagher et al., 2015; Harding et al., 2018). The third reference strain, DSM30011-VUB, is an environmental isolate (Repizo et al., 2017).

**Figure 2:**
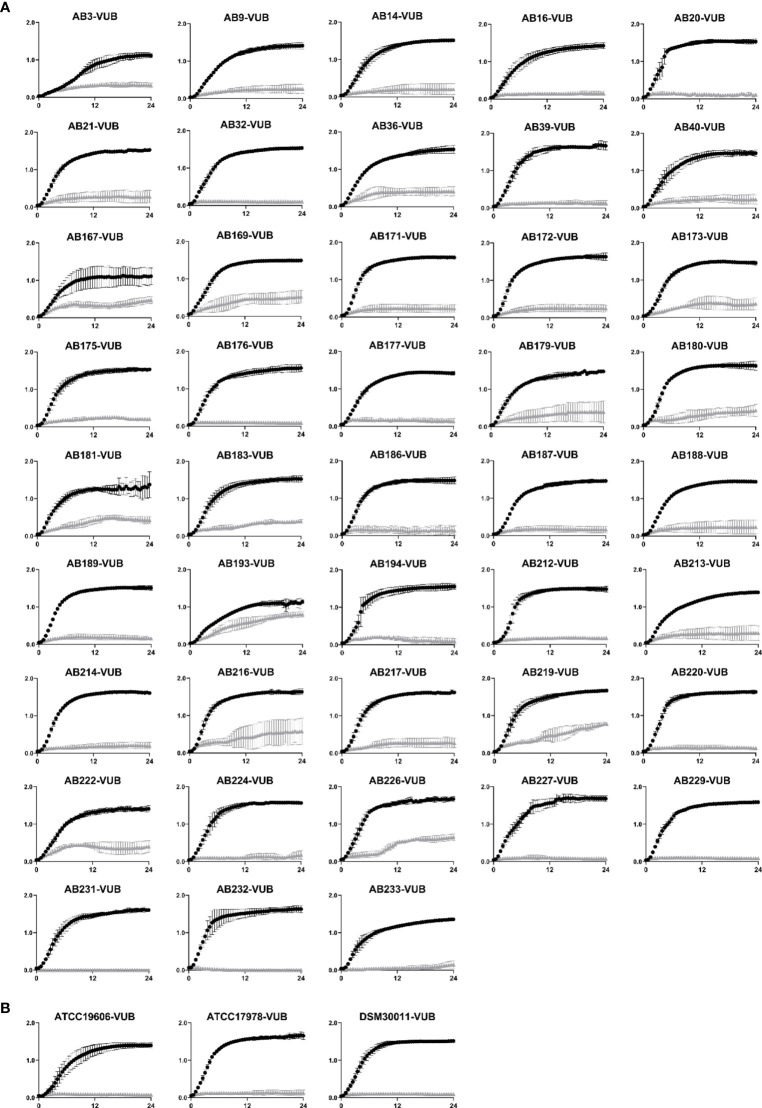
**(A)** Growth curves of 43 clinical *A. baumannii* isolates. **(B)** Growth curves of three *A*. *baumannii* reference strains (ATCC19606-VUB, ATCC17978-VUB and DSM30011-VUB). All strains were incubated with 100 µM of HDC1 (grey triangles) or without HDC1 (black spheres) for 24 h. All data points are shown as mean ± standard deviation of three independent biological replicates. X axis and y axis correspond to respectively time (h) and absorbance values measured at 600 nm.

The growth of all tested strains was impaired by the presence of HDC1 (**Figure 2**), with low, intermediate, or complete inhibition levels. An overview of the different resistance profiles against HDC1 can be found in **Table 1**. Complete inhibition of growth is observed for all three reference strains and 13 recent isolates, while most of the clinical isolates show an intermediate inhibition profile. The AB193-VUB isolate showed the least sensitivity to HDC1. No correlation could be established between the sensitivity of the tested strains to the HDC1 compound, and the presence of the antibiotic-resistance genes found in the modern clinical isolates tested.

**Table 1:** Growth inhibition levels on three reference strains and 43 clinical isolates of *A. baumannii*.

Level of growth inhibition		
Low	Intermediate	Complete
AB193-VUB	AB3-VUB	ATCC19606-VUB
	AB9-VUB	ATCC17978-VUB
	AB14-VUB	DSM30011-VUB
	AB16-VUB	AB20-VUB
	AB21-VUB	AB32-VUB
	AB36-VUB	AB39-VUB
	AB40-VUB	AB176-VUB
	AB167-VUB	AB177-VUB
	AB169-VUB	AB186-VUB
	AB171-VUB	AB189-VUB
	AB172-VUB	AB194-VUB
	AB173-VUB	AB220-VUB
	AB175-VUB	AB227-VUB
	AB179-VUB	AB229-VUB
	AB180-VUB	AB231-VUB
	AB181-VUB	AB232-VUB
	AB183-VUB	
	AB187-VUB	
	AB188-VUB	
	AB212-VUB	
	AB213-VUB	
	AB214-VUB	
	AB216-VUB	
	AB217-VUB	
	AB219-VUB	
	AB222-VUB	
	AB224-VUB	
	AB226-VUB	
	AB233-VUB	

The classification of strains into inhibition levels is based on the ratio of the final absorbance value of the HDC1-treated bacteria (OD_600nm; HDC1_) compared to the final absorbance value of the non-treated control (OD_600nm; control_) as followed: (i) low inhibition (OD_600nm; HDC1_ > 50% of OD_600nm; control_); (ii) intermediate inhibition (10% of OD_600nm; control_ < OD_600nm; HDC1_ < or = 50% of OD_600nm; control_) and (iii) complete inhibition (OD_600nm; HDC1_ < or = 10% OD_600nm; control_). The data were measured in biological triplicate.

## Discussion

In this study, a repurposing drug screen led to the discovery of a compound with inhibitory activity on the growth of a multidrug-resistant *Acinetobacter baumannii* reference strain, AB5075-VUB. This compound, named HDC1, is a harmine-derivative previously designed to have anticancer properties. The anticancer screen showed that the compound acts as a protein synthesis inhibitor at a concentration of 0.7 µM (Frédérick et al., 2012). In our study, HDC1 was shown to have bactericidal activity on AB5075-VUB with an IC_50_ of 48.23 µM. As the compound was previously found to be cytotoxic in HEPG2 cells at 50 µM, this limits the potential of HDC1 as a new antimicrobial without further modification of the molecule (Marx et al., 2019). Differences in active concentrations of HDC1 between cancer and bacterial cells could be attributed to cell size and target(s) presence and/or abundance, altered metabolism states and the presence or absence of post-translational modifications. However, as multidrug-, extensively drug- and pandrug-resistant *A. baumannii* strains are emerging and spreading, every option deems to be explored.

Due to the high diversity between *A. baumannii* isolates, one of the main hurdles in the discovery of new compounds is to find compounds capable of targeting the majority of the isolates. Here, we report a compound to have inhibitory activity on all the tested and recent carbapenem-resistant *A. baumannii* isolates. Our test shows various degrees of growth inhibition: from complete to intermediate to only slight inhibition. In addition, the 4 reference strains used in our study all show a high degree of sensitivity to HDC1. Taken together, this raises the following questions (i) what contributes to this difference in growth inhibition levels, (ii) what could be the target(s) of HDC1 and (iii) why is the whole AB5075-VUB bacterial population not killed by HDC1 in the tested conditions, since a significant bactericidal effect is observed? The phase variation observed in AB5075 might be the answer to the last question. Phase variation is a morphological feature of *A. baumannii* colonies where 2 different opacity phenotypes are observed: opaque and translucent (Tipton et al., 2015). Opaque and translucent colonies exhibit multiple phenotypic differences, including cell morphology, surface motility, biofilm formation and virulence, but of particular interest also a difference in resistance to antibiotics and thus potentially to HDC1 (Tipton et al., 2015; Chin et al., 2018). Interestingly, a recent study showed that HDC1 inhibits the phosphoserine phosphatase of *Mycobacterium tuberculosis*, MtSerB2 (Pierson et al., 2020). MtSerB2 catalyzes the last step in the L-serine biosynthetic pathway and is involved in immune evasion mechanisms of *M. tuberculosis* (Pierson et al., 2020). In AB5075-VUB, a homolog of MtSerB2 is present: AbSerB. This indicates a putative target of HDC1 in *A. baumannii*. A possible explanation for the different growth inhibition profiles of the clinical isolates could be the presence of mutations in the *serB* gene. Although *serB* is highly conserved in all our tested *A. baumannii* strains (sequence identity between 98.45-100%), no correlation could be found between mutations in *serB* and the growth inhibition profiles of the *A. baumannii* isolates (**Supplementary Table 2**). The target(s) of HDC1 in *A. baumannii* remain(s) to be determined. Interestingly, it has been shown for *M. tuberculosis* that certain compounds are more efficient at inhibiting growth of the bacterium itself, than inhibiting the enzyme only, suggesting different mechanisms of action or intracellular accumulation of the compounds (Haufroid and Wouters, 2019). Additional resistance mechanisms, potentially countering such effects, could explain the higher resistance to HDC1 of some clinical isolates. Nevertheless, HDC1 has broad activity on all the tested recent clinical *A. baumannii* isolates of our study, which prompts the further exploration of this compound and/or cognate putative target in *A. baumannii* for drug discovery.

## Conclusion

In conclusion, HDC1 is a potent harmine-derived compound with antibacterial activity identified using a multidrug-resistant *A. baumannii* strain, that also significantly inhibits the growth of diverse, recent, and carbapenem-resistant clinical *A. baumannii* isolates.

## Data Availability Statement

The original contributions presented in the study are included in the article/**Supplementary Material**. Further inquiries can be directed to the corresponding author.

## Author Contributions

Drafting of the manuscript, AB and CV. Corrections of the manuscript, AB, MV, ME, CP, JW, SB, and CV. HDC1 production, ME and SB. Preliminary drug screen, TQ, CP, and CV. Bioinformatics, AB and CH. Experiments, AB, CW, and CV. Data analyses, AB, MV, JW, and CV. All authors contributed to the article and approved the submitted version.

## Funding

AB is recipient of a PhD fellowship Strategic Basic Research of the Research Foundation – Flanders (FWO, File number: 77258). ME and SB acknowledge financial support of the Research Council at the Vrije Universiteit Brussel (VUB) through the IRP funding scheme. CV acknowledges the financial support from the Flanders Institute for Biotechnology (VIB). This project has received funding from the European Union’s Horizon 2020 research and innovation program under the Marie Sklodowska-Curie grant agreement No 748032.

## Conflict of Interest

The authors declare that the research was conducted in the absence of any commercial or financial relationships that could be construed as a potential conflict of interest.

## Publisher’s Note

All claims expressed in this article are solely those of the authors and do not necessarily represent those of their affiliated organizations, or those of the publisher, the editors and the reviewers. Any product that may be evaluated in this article, or claim that may be made by its manufacturer, is not guaranteed or endorsed by the publisher.

## Supplementary Material

Supplementary Table 1 Relationship between the absorbance (OD_600nm_) and the colony forming units (CFUs) per ml for the strain AB5075-VUB. The values are shown for overnight cultures that were diluted for accurate absorbance measurements. Rep = Biological replicate. At an OD_600nm_=1, the CFU/ml corresponds to 3.2 ± 0.3 10^8^ CFU/ml.

Supplementary Table 2 Percentage of nucleotide identity of the *serB* gene in 43 clinical isolates and four reference strains compared to the reference AB5075-UW (NZ_CP008706.1) with AA substitution where applicable.
