# Supplementary material for: Antimicrobial Activity of a Repurposed Harmine-Derived Compound on Carbapenem-Resistant Acinetobacter baumannii Clinical Isolates
Source: Front Cell Infect Microbiol. 2022 Jan 24;11:789672. doi: 10.3389/fcimb.2021.789672 (PMC8819726; doi:10.3389/fcimb.2021.789672)
Supplement: Supplementary Table 1 — Relationship between the absorbance (OD600nm) and the colony forming units (CFUs) per ml for the strain AB5075-VUB. The values are shown for overnight cultures that were diluted for accurate absorbance measurements. Rep = Biological replicate. At an OD600nm=1, the CFU/ml corresponds to 3.2 ± 0.3 108 CFU/ml. [file Table_1.docx]

**Supplementary data**

**Table S1**. Relationship between the absorbance (OD_600nm_) and the colony forming units (CFUs) per ml for the strain AB5075-VUB. The values are shown for overnight cultures that were diluted for accurate absorbance measurements. Rep = Biological replicate. At an OD_600nm_=1, the CFU/ml corresponds to 3.2$\pm$0.3 10^8^ CFU/ml.

|  | **Rep 1** | **Rep 2** | **Rep 3** | **Average** | **SD** |
| --- | --- | --- | --- | --- | --- |
| **OD_600_** | 6.4 | 6.5 | 6.3 | 6.4 | 0.1 |
| **CFU/ml** | 1.92 10^9^ | 2.06 10^9^ | 2.23 10^9^ | 2.07 10^9^ | 1.55 10^8^ |

**Table S2.** Percentage of nucleotide identity of the *serB* gene in 43 clinical isolates and four reference strains compared to the reference AB5075-UW (NZ_CP008706.1) with AA substitution where applicable.

| **Strain** | **Coverage (%)** | **Identity (%)** | **AA substitution** |
| --- | --- | --- | --- |
| AB3-VUB | 100.0 | 98.78 |  |
| AB9-VUB | 100.0 | 100.0 |  |
| AB14-VUB | 100.0 | 100.0 |  |
| AB16-VUB | 100.0 | 98.78 |  |
| AB20-VUB | 100.0 | 100.0 |  |
| AB21-VUB | 100.0 | 99.27 |  |
| AB32-VUB | 100.0 | 98.53 | V244I |
| AB36-VUB | 100.0 | 98.78 |  |
| AB39-VUB | 100.0 | 98.78 |  |
| AB40-VUB | 100.0 | 99.27 |  |
| AB167-VUB | 100.0 | 98.78 |  |
| AB169-VUB | 100.0 | 100.0 |  |
| AB171-VUB | 100.0 | 98.78 |  |
| AB172-VUB | 100.0 | 98.78 |  |
| AB173-VUB | 100.0 | 98.78 |  |
| AB175-VUB | 100.0 | 98.78 |  |
| AB176-VUB | 100.0 | 100.0 |  |
| AB177-VUB | 100.0 | 98.45 |  |
| AB179-VUB | 100.0 | 99.59 |  |
| AB180-VUB | 100.0 | 98.78 |  |
| AB181-VUB | 100.0 | 98.61 |  |
| AB183-VUB | 100.0 | 98.78 |  |
| AB186-VUB | 100.0 | 98.45 |  |
| AB187-VUB | 100.0 | 100.0 |  |
| AB188-VUB | 100.0 | 100.0 |  |
| AB189-VUB | 100.0 | 99.02 |  |
| AB193-VUB | 100.0 | 98.78 |  |
| AB194-VUB | 100.0 | 98.78 |  |
| AB212-VUB | 100.0 | 98.78 |  |
| AB213-VUB | 100.0 | 98.78 |  |
| AB214-VUB | 100.0 | 98.78 |  |
| AB216-VUB | 100.0 | 98.78 |  |
| AB217-VUB | 100.0 | 98.78 |  |
| AB219-VUB | 100.0 | 98.78 |  |
| AB220-VUB | 100.0 | 98.78 |  |
| AB222-VUB | 100.0 | 99.02 |  |
| AB224-VUB | 100.0 | 98.78 |  |
| AB226-VUB | 100.0 | 98.78 |  |
| AB227-VUB | 100.0 | 100.0 |  |
| AB229-VUB | 100.0 | 100.0 |  |
| AB231-VUB | 100.0 | 98.61 |  |
| AB232-VUB | 100.0 | 100.0 |  |
| AB233-VUB | 100.0 | 98.78 |  |
| AB5075-VUB | 100.0 | 100.0 |  |
| ATCC17978-VUB | 100.0 | 98.86 |  |
| ATCC19606-VUB | 100.0 | 98.45 |  |
| DSM30011-VUB | 100.0 | 98.86 | A340V |
